# Profiles of Impulsivity in Problematic Internet Users and Cigarette Smokers

**DOI:** 10.3389/fpsyg.2019.00772

**Published:** 2019-04-04

**Authors:** Su-Jiao Liu, Yan Lan, Lin Wu, Wan-Sen Yan

**Affiliations:** ^1^Department of Psychology, School of Philosophy and Sociology, Jilin University, Changchun, China; ^2^Department of Psychology, School of Medical Humanitarians, Guizhou Medical University, Guiyang, China; ^3^Department of Sociology, Wuhan University, Wuhan, China

**Keywords:** problematic internet use, smoking, addiction, impulsivity, personality

## Abstract

Problematic Internet use (PIU) has been gradually recognized as a mental health issue among adolescents and young students. PIU shows many similarities with substance use disorders, but the shared and distinct mechanisms underlying them are unclear. The purpose of the current study was to explore the relationships between impulsive traits and PIU as well as cigarette smoking behaviors among young adults. Two independent samples of university students (*N*_1_ = 1281, *N*_2_ = 1034, respectively) over 3 years were assessed with multiple measurements of impulsivity, including the Barratt Impulsiveness Scale-11 (BIS-11), the UPPSP Impulsive Behaviors Scale (UPPSP), and the Delay-discounting Test (DDT). Logistic regression models revealed that across the two independent samples, BIS-11 Attentional Impulsiveness was the common trait positively predicting both PIU and cigarette smoking. While BIS-11 Motor Impulsiveness as well as UPPSP Lack of Perseverance, Lack of Premeditation, and Negative Urgency were the typical traits linked to PIU as positive predictors, UPPSP Sensation Seeking was the unique trait linked to cigarette smoking as a positive predictor. These results suggested that specific dimensions of impulsivity might be concurrently implicated in PIU and cigarette smoking among young adults, putatively representing important trait marks for addictive behaviors.

## Introduction

Problematic Internet use or pathological Internet use (PIU), also regarded as Internet addiction (IA), is defined as an inability of individuals to control use of the Internet with various psychological and social problems (Young, 1998; Davis, 2001). Prevalence estimates of PIU in adolescents range from 1.0 to 9.0% among United States and European samples and from 2.0 to 18.0% in Asian samples (Spada, 2014). In the latest edition of the Diagnostic and Statistical Manual of Mental Disorders (i.e., DSM-5), Internet gaming disorder (IGD), a most prevalent form of PIU, has been included as a condition that requires future research in order to be considered a full disorder (American Psychiatric Association, 2013). Moreover, together with gambling disorder, IGD has been proposed in the list of addictive conditions in the latest revised version of the International Classification of Diseases (i.e., ICD-11; Potenza, 2018).

As a putative behavioral addiction similar with gambling disorder, PIU shares many clinical manifestations with substance use disorders (SUD), including excessive use of the Internet (e.g., excessive gaming and sexual preoccupations) with a loss of time sense, withdrawal symptoms (e.g., feelings of anger, tension, and depression), tolerance, and negative repercussions (Block, 2008). However, it is highly controversial whether PIU should be formally considered a new clinical disorder (Petry and O’brien, 2013), and more empirical studies are required to detect the shared and distinct aspects and mechanisms between PIU and SUD for a better comprehending of the nature of PIU.

Impulsivity is a multifaceted trait that is known as a characteristic construct of many mental disorders including addictive behaviors (Verdejo-García et al., 2008; De Wit, 2009; Meda et al., 2009), although its underlying neurobiological bases (e.g., impulsive disinhibition/decreased inhibitory control, impulsive inattention, impulsive decision-making, delay discounting) are highly heterogeneous (Winstanley et al., 2006; Reynolds et al., 2008). Measurement of impulsivity includes self-report personality questionnaires and laboratory-based cognitive tasks. For the former, the Barratt Impulsiveness Scale (BIS-11; Patton et al., 1995) measures the three-dimension model of impulsiveness (i.e., non-planning impulsiveness, attentional impulsiveness, and motor impulsiveness), and the UPPSP Impulsive Behaviors Scale (UPPSP; Whiteside and Lynam, 2001; Smith et al., 2007) figures five distinct pathways to impulsive behavior (i.e., negative urgency, positive urgency, lack of premeditation, lack of perseverance, and sensation seeking). For the latter, the Stop-Signal Test (Logan et al., 1997), Stroop Task (MacLeod, 1991), and analogous Go/No-Go Task mainly tax inhibitory control and response inhibition. Besides, the Delay-discounting Test (DDT; Kirby et al., 1999) assesses the discounting degree of delayed values when individuals make a choice between immediate and delayed rewards. The close connections of impulsivity with addictive behaviors have been well recognized in plentiful previous studies. Higher impulsivity traits measured by self-report questionnaires (e.g., the BIS-11) and impaired inhibitory control measured by cognitive tasks (e.g., the Stop-Signal Test) have been found in different forms of substance use disorders as well as in gambling disorder (Dick et al., 2010; Albein-Urios et al., 2012; Leeman and Potenza, 2012). Consistently, many studies have also revealed dysfunctional impulsivity properties in PIU on the BIS-11 (Cao et al., 2007; Lin et al., 2011; Dalbudak et al., 2013; Ryu et al., 2018), and on the Stop-Signal Test and Go/No-Go Task (Dong et al., 2010; Choi et al., 2014; Ding et al., 2014). Particularly, PIU subjects showed increased impulsivity traits comparable to patients with gambling disorder on the BIS-11 (Lee et al., 2012). These findings signified the candidacy of impulsivity as a vulnerability marker for different addictive disorders, and impulsivity might also serve as an aspect in identifying the nature of PIU as a potential addiction (Lee et al., 2012; Grant and Chamberlain, 2014). However, little research has directly compared PIU with substance use disorders (SUD) on impulsivity, and the shared and distinct mechanisms underlying them remain unclear. The present study thus aimed to investigate the associations of impulsivity traits with PIU and one usual type of SUD, cigarette smoking, among Chinese college students.

In contrast to other segments of society, young adult college students are believed to be more involved in PIU, because of their easy access to the Internet and rapid psychological and biological challenges during the late adolescence and young adulthood (Kandell, 1998; Li et al., 2010). According to the 42nd China Statistical Report on Internet Development 2018 by the China Internet Network Information Center (CNNIC), there are 802 million Internet users in China, of which 24.8% were young students and 27.9% were aged 20–29. These youths (so-called post-1990s generation), unlike their predecessors who are considered introverted, submissive, self-restrained, and responsible, have been labeled extroversive, promiscuous, imprudent, and impulsive as the economic wealth exploded prosperously (Rosen, 2009; Yang and Zheng, 2012), which might pave the way for a greater risk of PIU. Indeed, addictive Internet use among Chinese adolescents and college students has been found to be connected with higher levels of impulsivity measured by the BIS-11 (Cao et al., 2007; Wu et al., 2013; Zhang et al., 2015; Du et al., 2016), the Go/No-Go Task (Dong et al., 2010; Ding et al., 2014), and the DDT (Li et al., 2016; Tian et al., 2018). Interestingly, gender differences on the prevalence of PIU were evident among younger adolescent students, with a higher rate in males (Sun et al., 2012; Wang et al., 2013; Liang et al., 2016); however, this tendency appeared to vanish between males and females in adult college students (Ni et al., 2009; Yan et al., 2014; Yang et al., 2016; Nie et al., 2017). Although females are traditionally recognized as less impulsive and more compliant than males in China, and are less likely being active games players than males (Li and Kirkup, 2007), they could still plunge into impulsive online shopping and network communication activities in college (Wang et al., 2012), which might partly account for the vanished differences.

Analogous to the Internet use, cigarette smoking, which is not prohibited by law in most Western countries as well as in China, is accessible for youths and has been increasing among students from middle and high schools to universities (Alexander et al., 2001; Chen et al., 2004; Reed et al., 2007; Centers for Disease Control and Prevention [CDC], 2012). The prevalence of cigarette smoking was very high in Chinese adults aged 35–74 years, with 60.2% of men and 6.9% of women being current cigarette smokers (Gu et al., 2004). The Chinese college students are also holding a high risk for cigarette smoking, and the overall prevalence of current smokers is about 29–29.8% (45.1–49% for males and 5–6% for females) (Chen et al., 2004; Zhu et al., 2004; Mao et al., 2008). Similar to PIU, cigarette smoking behavior has also been found to be related to impulsivity. Smokers are typically more impulsive than non-smokers on self-report personality measures (Mitchell, 1999; Reynolds et al., 2007; Flory and Manuck, 2009; Balevich et al., 2013; Schulte et al., 2017), as well as on behavioral choice tasks using the discounting paradigm (Bickel et al., 1999; Baker et al., 2003; Reynolds et al., 2004; Ohmura et al., 2005; Białaszek et al., 2017). A recent meta-analysis of the relationships between specific impulsivity traits and cigarette smoking showed that the UPPSP model of impulsivity was significantly associated with smoking status and severity of nicotine dependence in adults (Kale et al., 2018). Although cigarette smoking is considered a daily-life consuming behavior similar to the Internet use to some degree, it does possess the core addictive features as a typical category of SUD (e.g., intake of substance, toxic effects), which are not necessarily seen in PIU. Therefore, a direct comparison between cigarette smoking and PIU on impulsivity might be conducive to dissociating the effects of substance use (i.e., nicotine) on impulsivity traits from the impulsive vulnerabilities that predispose these problematic behaviors (Verdejo-García et al., 2008). It is assumed that vulnerability-related impulsive aspects could be present in both behaviors, whereas specific impulsivity traits associated with nicotine use might only exist in cigarette smoking but not in PIU.

In the current study, we directly compared the impulsivity profiles between problematic Internet users and cigarette smokers in a large cross-sectional sample of Chinese college students by using the Barratt Impulsiveness Scale (BIS-11), the UPPSP Impulsive Behaviors Scale, and the Delay Discounting Test (DDT). Moreover, in view of the fast-growing popularity of the Internet information technologies (e.g., gaming, shopping, online payment, and social networking) among the new generation arising in China (i.e., post-1990s), we simultaneously included two independent samples across 3 years (selected in the year 2014 and 2017, respectively), so as to test the possible development of PIU and smoking behaviors among the young college students. In addition, previous reports have been discrepant on the relationships of impulsivity with PIU and smoking behaviors. For instance, some studies revealed robust associations of impulsivity traits (e.g., on the BIS-11 and DDT) with PIU (Saville et al., 2010; Lee et al., 2012; Li et al., 2016; Tian et al., 2018), whereas others failed to detect analogous connections (Rømer Thomsen et al., 2018; Zhou et al., 2018). Thus the inclusion of two separate data collections, which took place 3 years apart from each other, was also aimed at verifying the consistency of the relationships between impulsivity with PIU and smoking behaviors in the present study.

The objective of this study was twofold: (1) to facilitate the understanding of the nature of PIU by a straightforward comparison with cigarette smoking on impulsivity facets; and (2) to gather more empirical evidence about specific impulsivity-related traits linked to PIU and/or cigarette smoking as potential vulnerability candidates. We hypothesized that certain aspects of impulsivity (e.g., cognitive impulsiveness) might be commonly associated with PIU and smoking as potential vulnerability-related traits, while some domain-specific traits (e.g., sensation seeking, delay discounting) would be distinctly involved in each behavior, linked to PIU or cigarette smoking separately.

## Materials and Methods

### Participants and Procedure

The participants included two independent sample cohorts (i.e., Sample 1 and Sample 2) of young adult students from a local university located in Guiyang City, the capital of Guizhou Province, China. The Sample 1 consisted of 1,318 students, who were recruited from 12 randomly selected freshman classes at the university in November 2014. Similarly, the Sample 2 consisted of 1,060 students recruited from 10 randomly selected freshman classes at the same university in November 2017. As mentioned before, the purpose of recruiting these two separate samples was to investigate the possible over-time effects on PIU, smoking, and impulsivity.

All of these students were kindly invited to complete a series of questionnaires and provide demographic information by self-report in a psychology class lasting 45 min. The inclusion criteria included: (1) ≥18 years of age, and (2) willingness to participate in our study. The exclusion criteria included: (1) current or past major psychiatric disorders (e.g., schizophrenia, major depressive disorder, bipolar disorder), (2) a history of brain injury or trauma, neurological diseases, or mental disorders, and (3) ever or current use of psychoactive substance (e.g., opioids, cocaine, marijuana, and amphetamine), which were evaluated by self-report. There were 37 students in the Sample 1 and 26 students in the Sample 2 excluded from this study because of meeting one or more of these exclusion criteria.

Thus finally, 1,281 participants in the Sample 1 (aged 18–23 years, mean age = 19.1 ± 1.1 years; 434 males, 33.9%) and 1,034 participants in the Sample 2 (aged 18–23 years, mean age = 19.2 ± 1.1 years; 405 males, 39.2%) were included in this study. All subjects supplied written informed consent, and they were compensated with a gift equivalent to RMB ¥ 50 for their time. The Human Research Ethics Committee at the Guizhou Medical University thoroughly reviewed and approved this study. Our proposed study design, subject recruitment process, and our plans to compensate the participants were in accordance with the Declaration of Helsinki.

### Group Classification

The Internet use status was classified by employing the Chinese version of the Internet Addiction Test (IAT; Young, 1998), which has been carefully validated and widely used among Chinese students (e.g., Cao et al., 2011; Wang et al., 2011; Dong et al., 2012; Tang et al., 2014). The IAT is a self-report questionnaire with 20 items. Each item is rated on a 5-point Likert scale from 1 (not at all) to 5 (always). The IAT total score ranges from 20 to 100. Higher scores indicate a greater tendency of excessive Internet use symptoms. Scores ≥ 50 indicate potential problematic Internet use (PIU), while scores 20–49 indicate normal Internet use (NIU) (Khazaal et al., 2008). The Cronbach’s α was 0.92 (Sample 1) and 0.90 (Sample 2) in this study.

The smoking status was evaluated by using the Fagerström Test for Nicotine Dependence (FTND; Heatherton et al., 1991), which is a 6-item scale designed for measuring the degree of nicotine dependence. The total score on the FTND is 10, with higher scores indicating more nicotine use symptoms (Fagerström et al., 1996; Ríos-Bedoya et al., 2008). We adopted the Chinese version of the FTND, which has showed acceptable reliability and validity in previous research (e.g., Chen et al., 2002; Yang et al., 2011; Zhang et al., 2012). The Cronbach’s α was 0.86 (Sample 1) and 0.87 (Sample 2) in our study. Subjects were divided into subgroups according to their FTND scores and smoking history. Non-smokers were defined as persons who had never smoked cigarettes throughout their lifetime and scored 0 on the FTND. While smokers, including current and past smokers, were defined as persons who smoked at least one cigarette per day during past or current days and scored ≥ 1 on the FTND.

### Impulsivity Measures

We used the Barratt Impulsiveness Scale-11 (BIS-11; Patton et al., 1995) to measure three dimensions of impulsivity, including non-planning impulsiveness (a tendency to plan and think carelessly), attentional impulsiveness (refers to difficulties in focusing on a task and cognitive instability such as racing thoughts and thought insertion), and motor impulsiveness (a tendency to act on the spur of the moment). Each of the three dimensions consists of 10 items, with each item rated on a 4-point Likert scale. Sum scores of the three dimensions were obtained for further analyses, respectively, with higher scores indicating higher levels of impulsivity. In our study, the Chinese version of BIS-11 (Li et al., 2011) was adopted. The Cronbach’s α was 0.77–0.89 for the three subscales.

The UPPSP Impulsive Behaviors Scale (UPPSP; Whiteside and Lynam, 2001; Smith et al., 2007) was also used to evaluate five distinct personality traits of impulsive behavior (i.e., negative urgency, lack of premeditation, lack of perseverance, sensation seeking, and positive urgency). The UPPSP is a 59-item self-report inventory rated on a 4-point scale (1 = strongly agree, 4 = strongly disagree). The total score for each dimension was obtained for analyses. Higher scores indicate higher levels of impulsivity. The Chinese version of UPPSP has been used properly among college students in our previous study (Yan et al., 2016), and was employed in the present study. The Cronbach’s α was 0.75–0.84 for the five subscales.

The Delay-discounting Test (DDT; Kirby et al., 1999) was used to evaluate the discounting degree of delayed rewards in monetary choice. Subjects have to make a decision between a smaller but immediate reward and a larger but delayed reward. It has been supposed that delay discounting represents a tendency that subjects prefer the smaller immediate rewards to the larger delayed rewards (Dixon et al., 2003), signifying an impulsive behavior style in decision making. There are a set of 27 coupled choices on the DDT. The delay discounting degree was determined by using the hyperbolic equation *V* = *A*/(1 + *kD*), in which *k* is a free parameter describing the degree of discounting (see details in Kirby et al., 1999). A higher degree of delay discounting is described by a larger *k*-value. In our study, we adopted a culturally adapted Chinese version among college students (Sun and Li, 2011), which has been closely reported in our previous study (Yan et al., 2018). The *k*-values were calculated and log-transformed in order to get a normal distribution.

### Data Analysis

Data were administered and analyzed with the Statistical Package for the Social Sciences for Windows, Version 16.0 (SPSS Inc., Chicago, IL, United States). Differences on the categorical data (i.e., home locality, ethnicity, and gender) between groups were analyzed with chi-square tests. Scores on impulsivity measurements were compared between PIU and NIU groups with *t*-tests. As the group difference on gender was significant between smokers and non-smokers, the 2 (group: smokers, non-smokers) × 2 (gender: male, female) multivariate analysis of variance (mANOVA) model was employed to compare impulsivity scores of smokers and non-smokers. Impulsivity scores between non-smoking NIU, pure PIU, pure smoking, and smoking PIU subgroups were compared using the mANOVA model. *Post hoc* comparisons were analyzed with Fisher’s least significant differences protected *t*-tests. Partial correlations were tested between IAT/FTND scores and impulsivity measurements. Multiple linear regressions were employed to analyze the effects of impulsivity traits on IAT and FTND scores. Furthermore, logistic regression analyses were used to test the effects of different traits of impulsivity on PIU and smoking behaviors, controlling for gender. Multicollinearity was tested for all independent variables and outcome variables. Collinearity was not a problem for any variable according to the variance inflation factor (VIF < 10). Statistical significance was set as *p* < 0.05, two-tailed.

## Results

### Demographic Characteristics of the Sample 1 and Sample 2

For the purpose of investigating the possible development of problematic Internet use (PIU) and cigarette smoking conditions among young college students, the differences between Sample 1 and Sample 2 were tested on demographics, PIU and smoking status, and impulsivity scores. As seen in Table 1, there were no significant differences on age, ethnicity, and home locality between these two samples (*p*s > 0.05). However, the proportion of males in Sample 2 was moderately higher than that in Sample 1 (39.2% vs. 33.9%, χ^2^ = 6.925, *p* = 0.008). The average IAT and FTND scores, as well as the proportion of problematic Internet users (PIUs), smokers, and smoking PIUs, were comparable in the two samples (*p*s > 0.05).

**Table 1:** Demographic characteristics of the two samples.

Variables	Sample 1 (*N* = 1281)	Sample 2 (*N* = 1034)	*t*/χ^2^	*p*
Age, years (*M ± SD*)	19.09 ± 1.10	19.16 ± 1.05	–1.377	0.169
Gender, Male *n* (%)	434 (33.9)	405 (39.2)	6.925	0.008
Ethnicity, Hans *n* (%)	781 (61.0)	595 (57.5)	2.783	0.095
Home locality, Urban *n* (%)	300 (23.4)	230 (22.2)	0.448	0.503
IAT score (*M ± SD*)	36.77 ± 10.68	37.63 ± 10.19	–1.666	0.096
PIUs, *n* (%)	156 (12.2)	136 (13.2)	0.493	0.482
FTND score (*M ± SD*)	0.12 ± 0.65	0.16 ± 0.85	–1.103	0.270
Smokers, *n* (%)	61 (4.8)	48 (4.6)	0.018	0.892
Smoking PIUs, *n* (%)	7 (0.5)	5 (0.5)	0.044	0.834


*IAT, Internet Addiction Test; PIUs, problematic Internet users; FTND, Fagerström Test for Nicotine Dependence.*

### Profiles on Impulsivity of Problematic Internet Users

The demographics and impulsivity scores of the problematic Internet users (PIUs) were compared with the normal Internet users (NIUs) across two samples to present a general impulsivity profile of the PIUs, without excluding the confounding effects of smoking. As depicted in Table 2, no significant between-group differences were detected on age, gender, ethnicity, home locality, or smoking status (i.e., the FTND scores and the proportion of smokers) between PIUs and NIUs (*p*s > 0.05). On the BIS-11, PIUs scored higher than NIUs in both samples on Motor Impulsiveness (*t* = 8.494, *p* < 0.001, Cohen’s *d* = 0.48; *t* = 8.105, *p* < 0.001, Cohen’s *d* = 0.50, respectively), Attentional Impulsiveness (*t* = 9.677, *p* < 0.001, Cohen’s *d* = 0.54; *t* = 8.429, *p* < 0.001, Cohen’s *d* = 0.52, respectively), and Non-planning Impulsiveness (*t* = 7.114, *p* < 0.001, Cohen’s *d* = 0.40; *t* = 6.005, *p* < 0.001, Cohen’s *d* = 0.37, respectively). On the UPPSP, PIUs scored higher than NIUs in both samples on Lack of Perseverance (*t* = 7.114, *p* < 0.001, Cohen’s *d* = 0.40; *t* = 7.793, *p* < 0.001, Cohen’s *d* = 0.49, respectively), Lack of Premeditation (*t* = 3.640, *p* < 0.001, Cohen’s *d* = 0.20; *t* = 4.251, *p* < 0.001, Cohen’s *d* = 0.26, respectively), Negative Urgency (*t* = 8.601, *p* < 0.001, Cohen’s *d* = 0.48; *t* = 7.744, *p* < 0.001, Cohen’s *d* = 0.48, respectively), and Positive Urgency (*t* = 6.806, *p* < 0.001, Cohen’s *d* = 0.38; *t* = 5.993, *p* < 0.001, Cohen’s *d* = 0.37, respectively) except on Sensation Seeking (*p*s > 0.05). On the DDT *k*-values, PIUs did not differ from NIUs in any of the two samples (*p*s > 0.05).

**Table 2:** Demographic characteristics and impulsivity scores of PIUs and NIUs in each sample.

Variables	Sample 1 (*N* = 1281)				Sample 2 (*N* = 1034)			
	PIUs (*n* = 156)	NIUs (*n* = 1125)	*t*/χ^2^	*p*	PIUs (*n* = 136)	NIUs (*n* = 898)	*t*/χ^2^	*p*
Age, years (*M ± SD*)	19.01 ± 1.05	19.11 ± 1.11	–1.064	0.288	19.20 ± 1.05	19.15 ± 1.05	0.497	0.619
Gender, Male *n* (%)	57 (36.5)	377 (33.5)	0.561	0.454	56 (41.2)	349 (38.9)	0.265	0.607
Ethnicity, Hans *n* (%)	92 (59.0)	689 (61.2)	0.297	0.586	75 (55.1)	520 (57.9)	0.368	0.544
Home locality, Urban *n* (%)	45 (28.8)	255 (22.7)	2.917	0.088	34 (25.0)	196 (21.8)	0.688	0.407
IAT score (*M* ±*SD*)	57.83 ± 7.32	33.85 ± 7.24	38.725***	0.000	56.29 ± 6.90	34.81 ± 7.18	33.664***	0.000
FTND score (*M* ±*SD*)	0.14 ± 0.70	0.12 ± 0.65	0.345	0.730	0.15 ± 1.02	0.16 ± 0.82	–0.170	0.865
Smokers, *n* (%)	7 (4.5)	54 (4.8)	0.030	0.863	5 (3.7)	43 (4.8)	0.330	0.566
**BIS-11 score *(M* ± *SD)***								
Motor Impulsiveness	22.53 ± 3.35	20.17 ± 3.23	8.494***	0.000	22.55 ± 3.55	19.94 ± 3.22	8.105***	0.000
Attentional Impulsiveness	19.42 ± 3.38	16.74 ± 3.21	9.677***	0.000	19.09 ± 3.19	16.76 ± 2.97	8.429***	0.000
Non-planning Impulsiveness	31.37 ± 4.52	28.69 ± 4.39	7.114***	0.000	31.23 ± 4.49	28.85 ± 4.28	6.005***	0.000
**UPPSP score (*M* ± *SD*)**								
Sensation Seeking	28.37 ± 6.53	28.29 ± 6.43	0.154	0.878	28.20 ± 6.50	27.90 ± 6.32	0.515	0.606
Lack of Perseverance	23.14 ± 4.38	20.79 ± 3.80	7.114***	0.000	23.42 ± 4.23	20.76 ± 3.62	7.793***	0.000
Lack of Premeditation	23.95 ± 5.38	22.44 ± 4.76	3.640***	0.000	24.04 ± 5.04	22.22 ± 4.59	4.251***	0.000
Negative Urgency	30.08 ± 5.71	25.97 ± 5.58	8.601***	0.000	30.47 ± 5.34	26.45 ± 5.68	7.744***	0.000
Positive Urgency	31.59 ± 6.63	27.75 ± 6.60	6.806***	0.000	32.07 ± 6.71	28.41 ± 6.63	5.993***	0.000
**DDT score *(M* ± *SD)***								
*k*	0.31 ± 0.25	0.31 ± 0.25	0.110	0.912	0.29 ± 0.20	0.29 ± 0.21	–0.043	0.966
*k* (log-transformed)	–0.69 ± 0.45	–0.71 ± 0.48	0.537	0.592	–0.72 ± 0.46	–0.69 ± 0.43	–0.626	0.532


*PIUs, problematic Internet users; NIUs, normal Internet users; IAT, Internet Addiction Test; FTND, Fagerström Test for Nicotine Dependence; BIS, Barratt Impulsiveness Scale; UPPSP, UPPSP Impulsive Behaviors Scale; DDT, Delay-discounting Test, and k represents the discounting rate. ^∗∗∗^p < 0.001.*

### Profiles on Impulsivity of Cigarette Smokers

The demographics and impulsivity scores of the smokers were compared with the non-smokers across two samples to depict a general impulsivity profile of the smokers, without excluding the confounding effects of PIU. As seen in Table 3, no significant group differences were observed for age, ethnicity, home locality, or Internet use status (i.e., the IAT scores and the proportion of PIUs) between smokers and non-smokers (*p*s > 0.05). But the proportion of males was significantly higher in smokers than that in non-smokers for both samples (χ^2^ = 112.910, *p* < 0.001; χ^2^ = 62.938, *p* < 0.001, respectively). Thus in further analyses, gender was controlled as a between-group variable using the 2 (group: smokers, non-smokers) × 2 (gender: male, female) mANOVA models. On the BIS-11, smokers scored higher than non-smokers in both samples on Motor Impulsiveness [*F*_(1,1277)_ = 6.773, *p* = 0.009, $\eta_{p}^{2}$ = 0.018, Cohen’s *d* = 0.19; *F*_(1,1030)_ = 11.618, *p* = 0.001, $\eta_{p}^{2}$ = 0.021, Cohen’s *d* = 0.24, respectively] and Attentional Impulsiveness [*F*_(1,1277)_ = 5.134, *p* = 0.024, $\eta_{p}^{2}$ = 0.017, Cohen’s *d* = 0.22; *F*_(1,1030)_ = 4.327, *p* = 0.038, $\eta_{p}^{2}$ = 0.014, Cohen’s *d* = 0.19, respectively] except on Non-planning Impulsiveness [*F*_(1,1277)_ = 0.517, *p* = 0.472; *F*_(1,1030)_ = 0.403, *p* = 0.526, respectively]. On the UPPSP, smokers had higher scores than non-smokers in both samples on Sensation Seeking [*F*_(1,1277)_ = 5.867, *p* = 0.016, $\eta_{p}^{2}$ = 0.040, Cohen’s *d* = 0.15; *F*_(1,1030)_ = 4.886, *p* = 0.027, $\eta_{p}^{2}$ = 0.049, Cohen’s *d* = 0.23, respectively], but not on Lack of Perseverance [*F*_(1,1277)_ = 0.716, *p* = 0.398; *F*_(1,1030)_ = 0.166, *p* = 0.684, respectively], Lack of Premeditation [*F*_(1,1277)_ = 0.788, *p* = 0.375; *F*_(1,1030)_ = 0.088, *p* = 0.767, respectively], Negative Urgency [*F*_(1,1277)_ = 0.045, *p* = 0.832; *F*_(1,1030)_ = 0.416, *p* = 0.519, respectively], or Positive Urgency [*F*_(1,1277)_ = 0.881, *p* = 0.348; *F*_(1,1030)_ = 0.001, *p* = 0.973, respectively]. Besides, no significant main effects of gender or interaction effects of group × gender were found on the BIS-11 and UPPSP scores (*p*s > 0.05). There were no significant group differences between smokers and non-smokers on the DDT [*F*_(1,1277)_ = 1.302, *p* = 0.254; *F*_(1,1030)_ = 0.072, *p* = 0.789, respectively], nor main effects of gender or interaction effects of group × gender on the DDT across the two samples (*p*s > 0.05).

**Table 3:** Demographic characteristics and impulsivity scores of Smokers and Non-smokers in each sample.

Variables	Sample 1 (*N* = 1281)				Sample 2 (*N* = 1034)			
	Smokers (*n* = 61)	Non-smokers (*n* = 1220)	*t*/χ^2^	*p*	Smokers (*n* = 48)	Non-smokers (*n* = 986)	*t*/χ^2^	*p*
Age, years (*M* ±*SD*)	19.18 ± 1.13	19.09 ± 1.10	0.623	0.533	19.56 ± 1.13	19.14 ± 1.05	1.466	0.103
Gender, Male *n* (%)	59 (96.7)	375 (30.7)	112.91***	0.000	45 (93.8)	360 (36.5)	62.938***	0.000
Ethnicity, Hans *n* (%)	33 (54.1)	748 (61.3)	1.270	0.260	28 (58.3)	567 (57.5)	0.013	0.910
Home locality, Urban *n* (%)	8 (13.1)	292 (23.9)	3.792	0.051	9 (18.8)	221 (22.4)	0.355	0.551
FTND score (*M* ±*SD*)	2.61 ± 1.58	0.00 ± 0.00	12.852***	0.000	3.42 ± 2.13	0.00 ± 0.00	11.102***	0.000
IAT score (*M* ±*SD*)	37.92 ± 11.61	36.71 ± 10.63	0.863	0.388	36.06 ± 11.56	37.71 ± 10.12	–1.095	0.274
PIUs, *n* (%)	7 (11.5)	149 (12.2)	0.030	0.863	5 (10.4)	131 (13.3)	0.330	0.566
**BIS-11 score *(M* ± *SD)***								
Motor Impulsiveness	21.84 ± 3.30	20.39 ± 3.32	3.321**	0.001	21.96 ± 3.07	20.25 ± 3.39	3.874***	0.000
Attentional Impulsiveness	18.74 ± 3.07	16.98 ± 3.34	4.013***	0.000	18.38 ± 3.40	17.00 ± 3.07	3.005**	0.003
Non-planning Impulsiveness	30.23 ± 4.20	28.95 ± 4.50	2.172*	0.030	29.38 ± 4.96	29.15 ± 4.35	0.347	0.729
**UPPSP score (*M* ± *SD*)**								
Sensation Seeking	30.48 ± 5.83	28.19 ± 6.45	2.715**	0.007	31.19 ± 6.21	27.78 ± 6.31	3.657***	0.000
Lack of Perseverance	22.28 ± 3.22	21.01 ± 3.97	2.449*	0.014	21.29 ± 4.04	21.10 ± 3.80	0.336	0.737
Lack of Premeditation	23.05 ± 4.42	22.60 ± 4.89	0.696	0.487	22.94 ± 4.54	22.44 ± 4.69	0.725	0.468
Negative Urgency	28.31 ± 5.33	26.37 ± 5.76	2.573*	0.010	28.12 ± 5.59	26.93 ± 5.80	1.400	0.162
Positive Urgency	30.05 ± 7.57	28.12 ± 6.67	2.186*	0.029	30.98 ± 7.17	28.79 ± 6.71	2.203*	0.028
**DDT score *(M* ± *SD)***								
*k*	0.37 ± 0.25	0.30 ± 0.25	1.988*	0.047	0.30 ± 0.20	0.29 ± 0.21	0.429	0.668
*k* (log-transformed)	–0.60 ± 0.46	–0.71 ± 0.47	1.923	0.055	–0.68 ± 0.44	–0.71 ± 0.46	0.566	0.571


*FTND, Fagerström Test for Nicotine Dependence; IAT, Internet Addiction Test; PIUs, problematic Internet users; BIS, Barratt Impulsiveness Scale; UPPSP, UPPSP Impulsive Behaviors Scale; DDT, Delay-discounting Test, and k represents the discounting rate. ^∗^p < 0.05, ^∗∗^p < 0.01, ^∗∗∗^p < 0.001.*

### Comparison of Impulsivity Between Pure PIUs and Pure Smokers

In order to directly compare the impulsivity profiles of the pure PIUs and pure smokers by excluding the potential effects of comorbidity between PIU and smoking, we divided the subjects into four subgroups in each sample: non-smoking normal Internet users (NIUs), pure PIUs, pure smokers, and smoking PIUs. Supplementary Tables S1, S2 in the Supplementary Material displayed the demographics and impulsivity scores of these four subgroups in Sample 1 and Sample 2, respectively. Considering the few observations of smoking PIUs in each sample and the similar and comparable primary characteristics (e.g., age, ethnicity, home locality, Internet use status, smoking status, and impulsivity scores) of the two samples, we then pooled them together (*N* = 2315) for more reliable group comparisons. The Chow test for data pooling showed equal linear regression coefficients between impulsivity traits and IAT/FTND scores [*F*_(9,2297)_ = 1.305, *p* < 0.05 for IAT; *F*_(9,2297)_ = 1.418, *p* < 0.05 for FTND, respectively] on two data sets (i.e., Sample 1 and Sample 2). The merged data were displayed in Table 4.

**Table 4:** Impulsivity scores of Non-smoking NIUs, pure PIUs, pure Smokers, and Smoking PIUs.

Variables	Merged Sample (*N* = 2315)				*F/*χ^2^	*Post hoc* test (*p* < 0.05)
	(a) Non-smoking NIUs (*n* = 1926)	(b) Pure PIUs (*n* = 280)	(c) Pure Smokers (*n* = 97)	(d) Smoking PIUs (*n* = 12)		
Age, years (*M ± SD*)	19.12 ± 1.08	19.07 ± 1.04	19.31 ± 1.12	19.67 ± 1.30	2.208	–
Gender, Male *n* (%)	634 (32.9)	101 (36.1)	92 (94.8)	12 (100)	174.492***	a, b < c, d
Ethnicity, Hans *n* (%)	1152 (59.8)	163 (58.2)	57 (58.8)	8 (66.7)	3.696	–
Home locality, Urban *n* (%)	438 (22.7)	75 (26.8)	13 (13.4)	4 (33.3)	7.119	–
IAT score (*M* ±*SD*)	34.28 ± 7.18	56.96 ± 7.09	34.21 ± 8.12	60.50 ± 8.19	852.243***	a, c < b, d
FTND score (*M* ±*SD*)	0.00 ± 0.00	0.00 ± 0.00	2.90 ± 1.78	3.50 ± 2.61	1866.81***	a, b < c < d
**BIS-11 score *(M* ± *SD)***						
Motor Impulsiveness	20.00 ± 3.21	22.54 ± 3.46	21.32 ± 3.21	22.50 ± 3.15	59.928***	a < b, c, d
Attentional Impulsiveness	16.67 ± 3.08	19.25 ± 3.28	18.44 ± 3.15	19.67 ± 3.65	69.828***	a < b, c, d
Non-planning Impulsiveness	28.71 ± 4.33	31.33 ± 4.50	29.76 ± 4.55	30.58 ± 4.62	38.507***	a < b, c, d
**UPPSP score (*M* ± *SD*)**						
Sensation Seeking	28.00 ± 6.38	28.08 ± 6.46	30.47 ± 5.95	33.33 ± 5.84	17.342***	a, b < c < d
Lack of Perseverance	20.73 ± 3.72	23.29 ± 4.34	21.72 ± 3.64	22.83 ± 3.43	41.444***	a < b, c, d; c < b
Lack of Premeditation	22.31 ± 4.69	24.03 ± 5.31	23.00 ± 4.67	23.00 ± 2.13	13.558***	a < b
Negative Urgency	26.10 ± 5.63	30.20 ± 5.56	27.82 ± 5.37	31.50 ± 4.91	53.526***	a < b, c, d; c < b, d
Positive Urgency	27.94 ± 6.56	31.73 ± 6.70	30.04 ± 7.51	33.83 ± 5.37	31.484***	a < b, c, d; c < b, d
**DDT score *(M* ± *SD)***						
*k*	0.30 ± 0.24	0.29 ± 0.22	0.32 ± 0.23	0.48 ± 0.22	2.698*	a, b, c < d
*k* (log-transformed)	–0.72 ± 0.47	–0.70 ± 0.44	–0.66 ± 0.46	–0.37 ± 0.21	2.591*	a, b, c < d


*NIUs, normal Internet users; PIUs, problematic Internet users; BIS, Barratt Impulsiveness Scale; UPPSP, UPPSP Impulsive Behaviors Scale; DDT, Delay-discounting Test, and k represents the discounting rate. ^∗^p < 0.05, ^∗∗∗^p < 0.001.*

There were no significant group differences on age, ethnicity, or home locality between the four subgroups (*p*s > 0.05), while the proportion of males was significantly higher in pure smokers and smoking PIUs than that in non-smoking NIUs and PIUS (*p* < 0.001). Therefore, gender was controlled as a covariate in the mANOVA models, with the subgroup as the independent variable and impulsivity scores on the BIS-11, UPPSP, and DDT as the dependent variables. Significant between-group effects were displayed on BIS-11 Motor Impulsiveness [*F*_(3,2307)_ = 59.928, *p* < 0.001, $\eta_{p}^{2}$ = 0.072], Attentional Impulsiveness [*F*_(3,2307)_ = 69.828, *p* < 0.001, $\eta_{p}^{2}$ = 0.083], and Non-planning Impulsiveness [*F*_(3,2307)_ = 38.507, *p* < 0.001, $\eta_{p}^{2}$ = 0.048], as well as on UPPSP Sensation Seeking [*F*_(3,2307)_ = 17.342, *p* < 0.001, $\eta_{p}^{2}$ = 0.027], Lack of Perseverance [*F*_(3,2307)_ = 41.444, *p* < 0.001, $\eta_{p}^{2}$ = 0.051], Lack of Premeditation [*F*_(3,2307)_ = 13.558, *p* < 0.001, $\eta_{p}^{2}$ = 0.017], Negative Urgency [*F*_(3,2307)_ = 53.526, *p* < 0.001, $\eta_{p}^{2}$ = 0.065], and Positive Urgency [*F*_(3,2307)_ = 31.484, *p* < 0.001, $\eta_{p}^{2}$ = 0.039], but not on the DDT (*p* > 0.05). *Post hoc* comparisons were then tested on the BIS-11 and UPPSP.

On the BIS-11, pure PIUs scored higher than non-smoking NIUs on Motor Impulsiveness (*p* < 0.001, Cohen’s *d* = 0.76), Attentional Impulsiveness (*p* < 0.001, Cohen’s *d* = 0.81), and Non-planning Impulsiveness (*p* < 0.001, Cohen’s *d* = 0.59). Pure smokers also scored higher than non-smoking NIUs on Motor Impulsiveness (*p* < 0.001, Cohen’s *d* = 0.41), Attentional Impulsiveness (*p* < 0.001, Cohen’s *d* = 0.57), and Non-planning Impulsiveness (*p* < 0.001, Cohen’s *d* = 0.24). However, pure PIUs did not differ from pure smokers on any of these scores on the BIS-11 (*p*s > 0.05).

On the UPPSP, pure PIUs scored higher than non-smoking NIUs on Lack of Perseverance (*p* < 0.001, Cohen’s *d* = 0.63), Lack of Premeditation (*p* < 0.001, Cohen’s *d* = 0.34), Negative Urgency (*p* < 0.001, Cohen’s *d* = 0.73), and Positive Urgency (*p* < 0.001, Cohen’s *d* = 0.57), except on Sensation Seeking (*p* > 0.05). By contrast, pure smokers scored higher than non-smoking NIUs on Lack of Perseverance (*p* = 0.001, Cohen’s *d* = 0.27), Negative Urgency (*p* < 0.001, Cohen’s *d* = 0.31), Positive Urgency (*p* = 0.002, Cohen’s *d* = 0.30), and Sensation Seeking (*p* < 0.001, Cohen’s *d* = 0.40), except on Lack of Premeditation (*p* > 0.05). Nevertheless, no significant differences were indicated between pure PIUs and pure smokers on the UPPSP scores (*p*s > 0.05).

### Relationships Between Impulsivity Measures and IAT/FTND Scores

The relationships between IAT/FTND scores and impulsivity measures were tested with partial correlations, controlling for age, gender, ethnicity, and home locality. As described in Table 5, IAT scores were positively correlated with the BIS Motor Impulsiveness, Attentional Impulsiveness, Non-planning Impulsiveness, and UPPSP Lack of Perseverance, Lack of Premeditation, Negative Urgency, and Positive Urgency (*r*_p_ = 0.179–0.420, *p*s < 0.001), except UPPSP Sensation Seeking and DDT log-transformed *k*-value (*p*s > 0.05), across the two samples. FTND scores were positively associated with the BIS Motor Impulsiveness, Attentional Impulsiveness, Non-planning Impulsiveness, and UPPSP Sensation Seeking, Negative Urgency, and Positive Urgency (*r*_p_ = 0.059–0.366, *p*s < 0.05), except UPPSP Lack of Perseverance, Lack of Premeditation, and DDT log-transformed *k*-value (*p*s > 0.05) for both samples.

**Table 5:** Partial correlations (*r*_p_) between IAT scores, FTND scores and impulsivity measures in each sample.

Variables	1	2	3	4	5	6	7	8	9	10
(1) IAT score	–									
	–									
(2) FTND score	0.036	–								
	0.000									
(3) BIS Motor Impulsiveness	0.344***	0.095***	–							
	0.353***	0.066*								
(4) BIS Attentional Impulsiveness	0.420***	0.117***	0.526***	–						
	0.380***	0.085**	0.488***							
(5) BIS Non-planning Impulsiveness	0.304***	0.106**	0.466***	0.505***	–					
	0.260***	0.105**	0.413***	0.488***						
(6) UPPSP Sensation Seeking	0.009	0.361***	0.040	0.045	0.155***	–				
	0.037	0.366***	0.079*	0.088*	0.146***					
(7) UPPSP Lack of Perseverance	0.305***	0.063*	0.323***	0.480***	0.579***	0.182***	–			
	0.365***	0.031	0.334***	0.486***	0.540***	0.153***				
(8) UPPSP Lack of Premeditation	0.179***	0.010	0.372***	0.357***	0.594***	0.141***	0.631***	–		
	0.201***	0.059	0.383***	0.380***	0.554***	0.082**	0.608***			
(9) UPPSP Negative Urgency	0.387***	0.107***	0.441***	0.501***	0.436***	0.103***	0.326***	0.258***	–	
	0.366***	0.104***	0.476***	0.502***	0.389***	0.127***	0.357***	0.315***		
(10) UPPSP Positive Urgency	0.295***	0.059*	0.395***	0.388***	0.314***	0.229***	0.220***	0.175***	0.749***	–
	0.291***	0.060*	0.381***	0.381***	0.261***	0.262***	0.268***	0.216***	0.774***	
(11) DDT *k* (log-transformed)	0.019	0.020	0.048	0.004	0.003	0.049	0.005	0.062*	0.003	0.034
	0.010	0.010	0.012	0.040	0.030	0.010	0.017	0.010	0.025	0.006


*IAT, Internet Addiction Test; FTND, Fagerström Test for Nicotine Dependence; BIS, Barratt Impulsiveness Scale-11; UPPSP, UPPSP Impulsive Behaviors Scale; DDT, Delay-discounting Test, and k represents the discounting rate. Control variables: age, gender, ethnicity, and home locality. ^∗^p < 0.05, ^∗∗^p < 0.01, ^∗∗∗^p < 0.001. In each cell, the upper values are correlation coefficients for Sample 1 (N = 1281), and the bottom values are correlation coefficients for Sample 2 (N = 1034).*

### Predictive Effects of Impulsivity Traits on PIU and Cigarette Smoking

The multiple linear regressions were used to analyze the effects of impulsivity scores (i.e., BIS-11, UPPSP, and DDT) on the IAT and FTND scores. Gender was also entered as a predictor, given the significant group differences on gender. As shown in Table 6, BIS Motor Impulsiveness, Attentional Impulsiveness, and UPPSP Lack of Perseverance, Lack of Premeditation, and Negative Urgency were positive predictors of IAT scores, whereas gender (male), BIS Attentional Impulsiveness, and UPPSP Sensation Seeking were positive predictors of FTND scores across the two samples.

**Table 6:** Multiple linear regression analyses of impulsivity measures on IAT and FTND scores in each sample.

Models		Dependent Variable: IAT score		Dependent Variable: FTND score	
		Standardized Coefficients β	*t*	Standardized Coefficients β	*t*
Gender (Male = 1,		0.057	1.059	0.275	9.814***
Female = 0)		0.024	0.765	0.207	6.456***
BIS Motor		0.126	4.071***	0.037	1.101
Impulsiveness		0.172	5.035***	0.025	0.650
BIS Attentional		0.225	6.285***	0.102	2.746**
Impulsiveness		0.139	3.762***	0.126	4.103**
BIS Non-planning		0.022	0.884	0.093	2.288
Impulsiveness		0.020	0.558	0.011	0.259
UPPSP Sensation		0.009	0.319	0.271	9.872***
Seeking		0.021	0.778	0.254	8.960***
UPPSP Lack of		0.150	4.236***	0.054	1.666
Perseverance		0.273	7.090***	0.074	1.813
UPPSP Lack of		0.103	3.122**	0.026	0.876
Premeditation		0.123	3.256**	0.052	1.254
UPPSP Negative		0.196	4.923***	0.083	1.877
Urgency		0.162	3.314***	0.009	0.187
UPPSP Positive		0.015	0.191	0.062	1.439
Urgency		0.011	0.133	0.007	0.111
DDT *k*		0.018	0.800	0.023	0.891
(log-transformed)		0.060	1.004	0.012	0.455
**Model Fits**	Sample 1	*F* = 33.071^∗∗∗^,		*F* = 10.353^∗∗∗^,	
		*R*^2^ = 0.253^∗∗∗^		*R*^2^ = 0.096^∗∗∗^	
	Sample 2	*F* = 30.212^∗∗∗^,		*F* = 5.613^∗∗∗^,	
		*R*^2^ = 0.245^∗∗∗^		*R*^2^ = 0.057^∗∗∗^	


*IAT, Internet Addiction Test; FTND, Fagerström Test for Nicotine Dependence; BIS, Barratt Impulsiveness Scale-11; UPPSP, UPPSP Impulsive Behaviors Scale; DDT, Delay-discounting Test, and k represents the discounting rate. ^∗∗^p < 0.01, ^∗∗∗^p < 0.001. In each cell, the upper values are coefficients for Sample 1 (N = 1281), and the bottom values are coefficients for Sample 2 (N = 1034).*

Furthermore, we also adopted logistic regression models to test the effects of impulsivity dimensions on PIU and smoking behaviors, by distinguishing pure PIUs and pure smokers from Non-smoking NIUs with specific impulsive traits (excluding the comorbidity between PIU and smoking). Two binary regression models were conducted to compare the matched subgroups in both samples, that is, pure PIUs vs. Non-smoking NIUs for PIU, and pure smokers vs. Non-smoking NIUs for cigarette smoking, not including the subgroup of smoking PIUs. The impulsivity dimensions and gender were entered as independent variables in the regression models.

Table 7 showed that in both samples, BIS Motor Impulsiveness (OR = 1.100, *p* < 0.01; OR = 1.133, *p* < 0.01, respectively), Attentional Impulsiveness (OR = 1.113, *p* < 0.01; OR = 1.107, *p* < 0.05, respectively), as well as UPPSP Lack of Perseverance (OR = 1.106, *p* < 0.01; OR = 1.142, *p* < 0.001, respectively), Lack of Premeditation (OR = 1.083, *p* < 0.01; OR = 1.072, *p* < 0.01, respectively), and Negative Urgency (OR = 1.062, *p* < 0.01; OR = 1.063, *p* < 0.01, respectively), positively predicted PIU in the pure PIUs vs. Non-smoking NIUs models (Nagelkerke *R*^2^ = 0.202, 0.209, respectively). By contrast, BIS Attentional Impulsiveness (OR = 1.125, *p* < 0.01; OR = 1.189, *p* < 0.01, respectively) and UPPSP Sensation Seeking (OR = 1.172, *p* < 0.001; OR = 1.123, *p* < 0.001, respectively) positively predicted cigarette smoking in the pure smokers vs. Non-smoking NIUs models (Nagelkerke *R*^2^ = 0.368, 0.282, respectively).

**Table 7:** Logistic regression analyses of impulsivity traits on PIU and smoking in each sample.

Models	Problematic Internet Use (PIU)^a^ (pure PIUs = 1 vs. Non-smoking NIUs = 0)			Cigarette Smoking^b^ (pure Smokers = 1 vs. Non-smoking NIUs = 0)		
	*B*	Wald χ^2^	OR (95% CI)	*B*	Wald χ^2^	OR (95% CI)
Gender (Male = 1, Female = 0)	0.417	4.214*	1.533 (1.020–2.304)	4.391	35.327***	80.460 (18.932–341.949)
	0.487	5.002*	1.645 (1.063–2.543)	3.583	30.912***	36.604 (10.289–130.229)
BIS Motor Impulsiveness	0.095	7.433**	1.100 (1.027–1.178)	0.108	3.342	1.115 (0.992–1.252)
	0.125	12.079**	1.133 (1.056–1.216)	0.092	2.570	1.106 (0.978–1.251)
BIS Attentional Impulsiveness	0.109	8.584**	1.113 (1.036–1.196)	0.117	9.476**	1.125 (1.063–1.271)
	0.100	5.616*	1.107 (1.018–1.204)	0.133	10.112**	1.189 (1.037–1.365)
BIS Non-planning Impulsiveness	0.038	1.587	1.040 (0.979–1.105)	0.073	1.969	1.075 (0.972–1.189)
	0.018	0.319	1.019 (0.955–1.086)	0.031	0.342	1.031 (0.932–1.140)
UPPSP Sensation Seeking	–0.004	0.072	0.996 (0.965–1.027)	0.156	12.745***	1.172 (1.091–1.299)
	0.003	0.015	1.002 (0.968–1.038)	0.119	9.806***	1.123 (1.071–1.263)
UPPSP Lack of Perseverance	0.102	9.608**	1.106 (1.038–1.179)	0.072	1.489	1.074 (0.958–1.204)
	0.133	13.028***	1.142 (1.063–1.228)	0.088	1.775	1.093 (0.988–1.253)
UPPSP Lack of Premeditation	0.070	7.190**	1.083 (1.014–1.125)	0.045	0.973	1.046 (0.878–1.264)
	0.068	6.576**	1.072 (1.010–1.129)	0.039	0.645	1.039 (0.946–1.145)
UPPSP Negative Urgency	0.060	5.384**	1.062 (1.009–1.118)	0.049	1.372	1.051 (0.967–1.142)
	0.061	5.391**	1.063 (1.012–1.120)	–0.009	0.027	0.991 (0.895–1.098)
UPPSP Positive Urgency	0.002	0.005	1.002 (0.962–1.043)	0.052	2.357	1.056 (0.888–1.015)
	0.000	0.000	1.000 (0.953–1.048)	0.043	1.186	1.045 (0.965–1.131)
DDT *k* (log-transformed)	0.080	0.156	1.084 (0.727–1.614)	0.033	0.800	1.040 (0.670–2.929)
	0.093	0.159	1.097 (0.696–1.730)	0.039	1.071	1.046 (0.919–1.194)


*PIUs, problematic Internet users; NIUs, normal Internet users; CI, confidence interval; OR, odds ratio; BIS, Barratt Impulsiveness Scale-11; UPPSP, UPPSP Impulsive Behaviors Scale; DDT, Delay-discounting Test, and k represents the discounting rate. ^∗^p < 0.05, ^∗∗^p < 0.01, ^∗∗∗^p < 0.001. ^**a**^Nagelkerke R^2^ = 0.202 in Sample 1 and Nagelkerke R^*2*^ = 0.209 in Sample 2. ^*b*^Nagelkerke R^*2*^ = 0.368 in Sample 1 and Nagelkerke R^2^ = 0.282 in Sample 2. In each cell, the upper values are coefficients for Sample 1 (Non-smoking NIUs, n = 1071; pure PIUs, n = 149; pure Smokers, n = 54), and the bottom values are coefficients for Sample 2 (Non-smoking NIUs, n = 855; pure PIUs, n = 131; pure Smokers, n = 43).*

## Discussion

This study depicted the profiles of impulsivity in problematic Internet use (PIU) and cigarette smoking across two independent samples of Chinese college students. Our data showed that pure problematic Internet users (PIUs) had elevated scores than non-smoking normal Internet users (NIUs) on the BIS-11 (Motor Impulsiveness, Attentional Impulsiveness, and Non-planning Impulsiveness) and on the UPPSP (Lack of Perseverance, Lack of Premeditation, Negative Urgency, and Positive Urgency). Pure cigarette smokers also scored higher than non-smoking NIUs on the BIS-11 (Motor Impulsiveness, Attentional Impulsiveness, and Non-planning Impulsiveness) and on the UPPSP (Lack of Perseverance, Negative Urgency, Positive Urgency, and Sensation Seeking). Significant positive correlations were found between IAT and FTND scores and most impulsivity traits on the BIS-11 and UPPSP. More interesting findings in this study were from the logistic regression models, revealing that BIS Attentional Impulsiveness was the common trait positively predicting both PIU and cigarette smoking. Additionally, although BIS Motor Impulsiveness and UPPSP Lack of Perseverance, Lack of Premeditation, and Negative Urgency characteristically predicted PIU, UPPSP Sensation Seeking predicted cigarette smoking uniquely. In particular, all of these findings were coherently detected across the two separate data collections (i.e., Sample 1 and Sample 2), which occurred 3 years apart from each other. As a whole, these results support our hypotheses that PIU as a candidate of addiction shares some basic mechanisms on impulsivity (Attentional Impulsiveness) with cigarette smoking, although each of these two problem behaviors could be characterized by linking to specific impulsive traits (i.e., Motor Impulsiveness, Lack of Perseverance, Lack of Premeditation, and Negative Urgency vs. Sensation Seeking), representing a commonality-specificity profile of impulsivity implicated in addiction.

Despite its multidimensional nature, impulsivity as a core pathological trait of addiction has been considered a vulnerability marker for substance use disorders (Verdejo-García et al., 2008; De Wit, 2009; Ersche et al., 2010; Robbins et al., 2012), playing a critical role in predicting onset and maintenance of drug taking and relapse rates (Pattij and De Vries, 2013). Impulsivity is also being regarded as a candidate for vulnerability to problematic Internet use (PIU) (Ryu et al., 2018), though PIU is controversially viewed as a behavioral addiction. In our study, problematic Internet users (PIUs) showed elevated scores on the BIS model of impulsive traits (i.e., Motor Impulsiveness, Attentional Impulsiveness, and Non-planning Impulsiveness), in keeping with previous studies (Cao et al., 2007; Lin et al., 2011; Dalbudak et al., 2013; Ryu et al., 2018); and they also exhibited higher levels on the UPPSP model (i.e., Lack of Perseverance, Lack of Premeditation, Negative Urgency, and Positive Urgency), despite that limited literature failed to find consistent associations between the UPPSP model and problematic Internet gaming/excessive online gaming (Irvine et al., 2013; Nuyens et al., 2016; Deleuze et al., 2017; Rømer Thomsen et al., 2018). Similarly, increased impulsivity traits were observed likewise in our cigarette smokers, both on the BIS model (i.e., Motor Impulsiveness, Attentional Impulsiveness, and Non-planning Impulsiveness) and on the UPPSP model (i.e., Sensation Seeking, Lack of Perseverance, Negative Urgency, and Positive Urgency), consistent with the literature of impulsivity in cigarette smoking (Kale et al., 2018). These findings indicated that PIUs showed an increased tendency on most impulsivity traits comparable to cigarette smokers (except Lack of Premeditation and Sensation Seeking), demonstrating the likelihood of PIU as a putative non-substance addiction (Block, 2008; Potenza, 2018). However, in our study PIUs and cigarette smokers did not differ from non-smoking NIUs on the DDT, inconsistent with previous reports showing a higher degree of delay discounting in adolescents with Internet gaming disorder (Saville et al., 2010; Wang et al., 2017; Tian et al., 2018) and in university students with cigarette smoking (Białaszek et al., 2017). Nevertheless, smoking PIUs in our study did have higher discounting rates (i.e., *k*-values) than non-smoking NIUs, PIUs, and smokers (Table 4). Divergence on these data might be partly due to different methodologies and samples, therefore universal measurements should be employed in future to investigate the discrepancy of results from different studies.

More importantly in this study, we found that BIS Attentional Impulsiveness positively predicted both problematic Internet use (PIU) and cigarette smoking in the logistic regression models, after excluding the confounding effects of comorbidity between PIU and smoking behaviors as well as demographic variables (i.e., gender). To our best knowledge, this finding presented the first direct evidence that specific trait of impulsivity (i.e., Attentional Impulsiveness) is overtly increased as a predictive indicator in both PIU and one typical form of substance use disorders (i.e., cigarette smoking) among young adult populations. This data, together with previous preliminary evidence revealing parallel increased impulsivity characteristics on the BIS model between PIU and gambling disorder (Lee et al., 2012), suggest that specific traits of impulsivity might serve as important vulnerability candidates for PIU (Grant and Chamberlain, 2014). These results support our hypothesis that certain impulsivity trait (i.e., Attentional Impulsiveness) is shared by both PIU and cigarette smoking behaviors, putatively representing the basic mechanism and marker for PIU and other addictive disorders. Furthermore, the logistic regression models showed that BIS Motor Impulsiveness and UPPSP Lack of Perseverance, Lack of Premeditation, and Negative Urgency predicted PIU, while UPPSP Sensation Seeking predicted cigarette smoking distinguishingly. This finding demonstrated a domain-specific tendency of impulsivity traits linked to PIU and cigarette smoking behaviors. Sensation Seeking refers to the liability to enjoy and pursue exciting activities that may be dangerous (Smith et al., 2007). It has been clearly showed that both male and female smokers were higher in Sensation Seeking than their non-smoking counterparts among adolescents and young adults (Carton et al., 1994; Martin et al., 2002), and Sensation Seeking had strong predictive value for cigarette use as well as marijuana and alcohol use in high school students (Crawford et al., 2003). Therefore, our data further support the notion that Sensation Seeking is an important factor in identifying youths at increased risk for smoking behaviors (Case et al., 2017). By contrast, Motor Impulsiveness (a tendency to act on the spur of the moment), Lack of Perseverance (refers to an ability to remain focused on a task), Lack of Premeditation (a tendency to think and reflect on the consequences of an act), and Negative Urgency (a tendency to experience strong impulses under the condition of negative affect) were indicated as the positive predictors for PIU separately. These results might potentially reflect the heightened risk of involving in PIU activities (e.g., gaming and shopping) for young adult students, who are dysfunctional in behavioral inhibition, myopic for the future, or having maladaptive emotion-focused coping styles (Hetzel-Riggin and Pritchard, 2011; Li et al., 2016; Nikolaidou et al., 2016).

Several limitations should be noted in this study. Firstly, the nature of our study design was a cross-sectional investigation essentially, despite the fact that we included two independent sample cohorts across 3 years (i.e., Sample 1 selected in 2014 and Sample 2 selected in 2017). Thus, the present study was not able to determine causal relationships of impulsivity traits with PIU and cigarette smoking behaviors and future longitudinal studies are warranted. Secondly, the Internet use and smoking status as well as impulsivity traits were primarily evaluated by using self-report questionnaires, which might be liable to bring subjective bias into data analyses. Therefore the results should be explained carefully, and more objective assessments should be incorporated in future research. Additionally, the self-report measurements (e.g., IAT and FTND) and our exclusion criteria for participants (e.g., current or past major psychiatric disorders) evaluated by self-report could suffer from social desirability bias, although we have tried to adopt anonymity and confidentiality methods to partially avoid this problem (e.g., the fear of stigma and stigmatization because of a history of psychiatric disorders among the young students). Thus, future similar research should include various methods (e.g., standard measures such as the Marlowe–Crowne Social Desirability Scale) to reduce social desirability bias and improve the reliability of data collected by self-reports. Thirdly, our samples mainly consisted of young adult university students, so the findings could not be generalized to the other populations of PIUs and smokers (e.g., community samples, treatment-seeking samples), neither to other age groups (e.g., middle and late adulthood). Moreover, the college students in our study were randomly selected from a single university located in the city of Guiyang, China, so that they could not be regarded as one of the nationally representative samples. Simultaneously, the specific Chinese context of our college students is quite different from that of most Western countries, which might also limit the generalizability of the study findings, thus future cross-cultural studies are warranted and should be of help to draw more universal conclusions on the relationship of impulsivity with PIU and smoking behaviors.

In despite of these limitations, our results indicated that problematic Internet use (PIU) as a potential addictive behavior shared the basic and common mechanism with smoking behavior on certain impulsivity trait (i.e., Attentional Impulsiveness), which is essentially relevant to maladaptive cognitive inhibition, including attention deficit (refers to difficulties in focusing on a task at hand) and cognitive instability (refers to thought insertion and racing thoughts) (Patton et al., 1995; Nigg, 2000). This finding provided the direct converged evidence in PIU and cigarette smoking, supporting the notion that impulsivity is a vulnerability marker for addictive disorders (Verdejo-García et al., 2008; De Wit, 2009; Ersche et al., 2010; Robbins et al., 2012; Pattij and De Vries, 2013). In addition, specific aspects of impulsivity were distinctively linked to PIU (i.e., Motor Impulsiveness, Lack of Perseverance, Lack of Premeditation, and Negative Urgency) and smoking (i.e., Sensation Seeking), representing a domain specificity of impulsive traits implicated in substance and non-substance addictions.

Our findings should be beneficial for future studies and probably throw light on the pathways to develop potential prevention methods and early interventions of different forms of addictive behaviors. For one thing, some cognitive training and behavioral interventions aimed at improving cognitive inhibition capacities that are related to Attentional Impulsiveness, such as working memory training and inhibitory control training, might be ecologically valid not only in pathological and clinical addictions (e.g., stimulants and alcohol dependence) (Houben et al., 2011; Wexler, 2011; Wiers et al., 2011; Wesley and Bickel, 2014), but also in less severe, non-clinical forms of problematic behaviors (e.g., PIU and cigarette smoking). Besides, more precise and effective training arrangements could be exploited by identifying specific targets of impulsivity (e.g., Attentional Impulsiveness) in further preclinical trials, especially for the youths who are also holding a high risk for other attention impulsivity-related disorders (e.g., attention deficit/hyperactivity disorder) (Evans et al., 2014). For another thing, the distinct patterns of impulsivity traits linked to PIU and cigarette smoking call for differentiated and personalized training schedules for individuals with varying types of addictions (i.e., substance use disorders vs. non-substance induced behavioral addictions) (Verdejo-García et al., 2008; Robbins et al., 2012; Yan et al., 2014). In this respect, typical facets of impulsivity should be targeted separately for the interventions in each condition (e.g., Negative Urgency for PIU, Sensation Seeking for cigarette smoking) among youths, who are not fully developed in brain cognition, and as such in emotional functioning. Especially in the Chinese cultural context, most children seem to be overindulged by their parents under the national one-child policy that is implemented since the 1980s and peaked in the 1990s in mainland China (Greenhalgh, 2003; Zhang and Goza, 2006); and school students in China are always suffering a heavy burden from the urgent expectancy of parents for their success in the college entrance examination. But paradoxically, most of these students could easily indulge themselves at university, staying away from parents and entrance exams. As a result, some mental disorders and behavioral disturbances (e.g., depression, anxiety, excessive gaming, smoking, shopping spree, binge eating and drinking) have been more and more salient in Chinese adolescents and young college students in recent decades (Liu and Zhou, 2002; Leung et al., 2008; Lian and Lin, 2008; Cao et al., 2011; Kieling et al., 2011; Yan et al., 2018). Our findings of the specific impulsivity traits involved in PIU (e.g., Negative Urgency) and cigarette smoking (e.g., Sensation Seeking), therefore, potentially designate a peculiar pathway to the prevention and intervention of certain problematic behaviors among Chinese college students. For instance, some school-based interventions (e.g., teaching techniques for coping and regulating negative affects) might help to reduce inappropriate catharsis of stress and prevent certain impulsive behaviors (e.g., PIU) related to negative urgency in these students (Berkman et al., 2012). By comparison, other behavioral trainings for resolving ambivalence and reducing the high levels of excitement pursuing, such as contingency management and motivational interviewing, could change subjective representations of rewards/losses and reduce the preference of college students for dangerous exciting activities connected with sensation seeking (e.g., smoking and drinking) (Hettema and Hendricks, 2010; Rohsenow et al., 2017). Last but not the least, different measures and models of impulsivity were utilized in our study, including a three-dimension model (i.e., the BIS-11) that mainly highlights cognitive-behavioral disinhibition facets (i.e., Attentional, Non-Planning, and Motor Impulsiveness) (Patton et al., 1995; Meda et al., 2009) and a five-pathway model (i.e., the UPPSP) that contains more diverse and complex traits (i.e., Negative Urgency, Positive Urgency, Lack of Perseverance, Lack of Premeditation, Sensation Seeking), as well as a delay discounting task (i.e., the DDT). However, because of the multi-dimensions and heterogeneity of these impulsivity traits, we failed to detect universal relationships between them with PIU and smoking, even though some traits seem to be similar in concept (e.g., Non-Planning Impulsiveness vs. Lack of Premeditation). Thus, our findings prompt that more integrated and refined models of impulsivity are quite necessary for future similar studies. As an example, a superior model that encompasses the all-round facets of impulsivity, including inhibitory control (e.g., cognitive inhibition related to Attentional Impulsiveness and Perseverance, behavioral inhibition related to Motor Impulsiveness), forward-looking planning (e.g., Delay Discounting, Premeditation, and Non-planning Impulsiveness), novelty/reward sensitivity (e.g., Sensation Seeking), and emotional urgency (e.g., Negative/Positive urgency), should be extremely beneficial to sniff out core marker traits of impulsivity implicated in addictive disorders, paving the way for better treating addiction in future (Ersche et al., 2010).

## Ethics Statement

The Human Research Ethics Committee at the Guizhou Medical University thoroughly reviewed and approved this study. Our proposed study design, subject recruitment process, and plans to compensate the participants were in accordance with the Declaration of Helsinki.

## Author Contributions

S-JL, LW, and W-SY designed the study, wrote the protocols, and directed the study. S-JL and W-SY wrote a first draft of the manuscript. S-JL and YL performed assessments, data collection, and main data analysis. All authors contributed to the writing and approved the final manuscript.

## Conflict of Interest Statement

The authors declare that the research was conducted in the absence of any commercial or financial relationships that could be construed as a potential conflict of interest.
