# Supplementary material for: Profiles of Impulsivity in Problematic Internet Users and Cigarette Smokers
Source: Front Psychol. 2019 Apr 4;10:772. doi: 10.3389/fpsyg.2019.00772 (PMC6458249; doi:10.3389/fpsyg.2019.00772)
Supplement: Supplementary file 1 [file Table_1.DOC]

Supplementary Material

**Table S1.** Impulsivity scores of Non-smoking NIUs, pure PIUs, pure Smokers, and Smoking PIUs in Sample 1.

| **Variables** | ***N*=1281** | | | | ***F/*χ^2^** | **Post-hoc test**  **(*p*<0.05)** |
| --- | --- | --- | --- | --- | --- | --- |
|  | **a. Non-smoking NIUs**  **(*n*=1071)** | **b. pure PIUs**  **(*n*=149)** | **c. pure Smokers**  **(*n*=54)** | **d. Smoking PIUs**  **(*n*=7)** |  |  |
| Age, years (*M±SD*) | 19.10±1.11 | 19.00±1.06 | 19.19±1.17 | 19.14±0.90 | 0.510 | - |
| Gender, Male n (%) | 325 (30.3) | 50 (33.6) | 52 (96.3) | 7 (100) | 113.552*** | a, b<c, d |
| Ethnicity, Hans n (%) | 659 (61.5) | 89 (59.7) | 30 (55.6) | 4 (57.1) | 1.868 | - |
| Home locality, Urban n (%) | 249 (23.2) | 43 (28.9) | 6 (11.1) | 2 (28.6) | 7.141 | - |
| IAT score (*M±SD*) | 33.79±7.20 | 57.66±7.28 | 34.87±7.93 | 61.43±8.00 | 501.219*** | a, c<b, d |
| FTND score (*M±SD*) | 0.00±0.00 | 0.00±0.00 | 2.54±1.61 | 3.14±1.35 | 1139.59*** | a, b<c<d |
| **BIS-11 score *(M±SD)*** |  |  |  |  |  |  |
| Motor Impulsiveness | 20.10±3.20 | 22.51±3.38 | 21.70±3.34 | 22.86±2.97 | 28.534*** | a<b, c, d |
| Attentional Impulsiveness | 16.65±3.19 | 19.40±3.40 | 18.59±3.05 | 19.86±3.24 | 37.945*** | a<b, c, d |
| Non-planning Impulsiveness | 28.62±4.39 | 31.34±4.57 | 30.02±4.26 | 31.86±3.48 | 18.676*** | a<b, c, d |
| **UPPSP score (*M±SD*)** |  |  |  |  |  |  |
| Sensation Seeking | 28.18±6.44 | 28.23±6.52 | 30.37±5.79 | 31.29±6.55 | 3.499* | a, b<c, d |
| Lack of Perseverance | 20.71±3.81 | 23.19±4.46 | 22.31±3.37 | 22.00±1.63 | 20.141*** | a<b, c, d |
| Lack of Premeditation | 22.41±4.77 | 23.99±5.49 | 23.06±4.67 | 23.00±1.63 | 4.810** | a<b |
| Negative Urgency | 25.87±5.58 | 30.00±5.78 | 27.87±5.37 | 31.71±3.68 | 27.168*** | a<b, c, d; c<b, d |
| Positive Urgency | 27.66±6.54 | 31.46±6.66 | 29.48±7.66 | 34.43±5.50 | 17.240*** | a<b, c, d; c<b, d |
| **DDT score *(M±SD)*** |  |  |  |  |  |  |
| *k* | 0.30±0.25 | 0.30±0.24 | 0.34±0.25 | 0.58±0.22 | 3.142* | a, b, c<d |
| *k* (log-transformed) | -0.72±0.48 | -0.71±0.45 | -0.64±0.46 | -0.27±0.17 | 2.507 | - |

Note. NIUs=normal Internet users, PIUs=problematic Internet users, IAT=Internet Addiction Test, FTND=Fagerström Test for Nicotine Dependence, BIS=Barratt Impulsiveness Scale, UPPSP=UPPSP Impulsive Behaviors Scale, DDT=Delay-discounting Test, and *k* represents the discounting rate. **p*<0.05, ***p*<0.01, ****p*<0.001.

**Table S2.** Impulsivity scores of Non-smoking NIUs, pure PIUs, pure Smokers, and Smoking PIUs in Sample 2.

| **Variables** | ***N*=1034** | | | | ***F/*χ^2^** | **Post-hoc test**  **(*p*<0.05)** |
| --- | --- | --- | --- | --- | --- | --- |
|  | **a. Non-smoking NIUs**  **(*n*=855)** | **b. pure PIUs**  **(*n*=131)** | **c. pure Smokers**  **(*n*=43)** | **d. Smoking PIUs**  **(*n*=5)** |  |  |
| Age, years (*M±SD*) | 19.13±1.05 | 19.15±1.01 | 19.47±1.05 | 19.40±1.02 | 1.237 | - |
| Gender, Male n (%) | 309 (36.1) | 51 (38.9) | 40 (93.0) | 5 (100) | 63.401*** | a, b<c, d |
| Ethnicity, Hans n (%) | 493 (57.7) | 74 (56.5) | 27 (62.8) | 4 (80.0) | 3.434 | - |
| Home locality, Urban n (%) | 189 (22.1) | 32 (24.4) | 7 (16.3) | 2 (40.0) | 2.167 | - |
| IAT score (*M±SD*) | 34.88±7.12 | 56.18±6.81 | 33.37±8.38 | 59.20±9.20 | 356.655*** | a, c<b, d |
| FTND score (*M±SD*) | 0.00±0.00 | 0.00±0.00 | 3.35±1.89 | 4.00±3.94 | 869.362*** | a, b<c<d |
| **BIS-11 score *(M±SD)*** |  |  |  |  |  |  |
| Motor Impulsiveness | 19.89±3.22 | 22.57±3.56 | 21.84±3.02 | 22.00±3.67 | 26.539*** | a<b, c, d |
| Attentional Impulsiveness | 16.69±2.94 | 19.08±3.15 | 18.26±3.29 | 19.40±4.56 | 27.689*** | a<b, c, d |
| Non-planning Impulsiveness | 28.82±4.25 | 31.32±4.43 | 29.94±4.93 | 31.80±5.81 | 12.865*** | a<b, c, d |
| **UPPSP score (*M±SD*)** |  |  |  |  |  |  |
| Sensation Seeking | 27.76±6.30 | 27.89±6.40 | 30.60±6.22 | 33.20±3.42 | 5.659** | a, b<c, d |
| Lack of Perseverance | 20.75±3.61 | 23.40±4.21 | 22.30±3.85 | 24.00±5.05 | 20.302*** | a<b, c, d |
| Lack of Premeditation | 22.18±4.58 | 24.08±5.10 | 22.93±4.72 | 23.00±2.92 | 6.457*** | a<b |
| Negative Urgency | 26.39±5.69 | 30.44±5.31 | 28.77±5.42 | 31.20±4.76 | 20.847*** | a<b, c, d; c<b, d |
| Positive Urgency | 28.29±6.57 | 32.03±6.76 | 30.74±7.34 | 33.00±5.70 | 13.922*** | a<b, c, d; c<b, d |
| **DDT score *(M±SD)*** |  |  |  |  |  |  |
| *k* | 0.29±0.21 | 0.29±0.20 | 0.30±0.21 | 0.34±0.14 | 0.108 | - |
| *k* (log-transformed) | -0.72±0.47 | -0.70±0.44 | -0.69±0.46 | -0.51±0.20 | 0.426 | - |

Note. NIUs=normal Internet users, PIUs=problematic Internet users, IAT=Internet Addiction Test, FTND=Fagerström Test for Nicotine Dependence, BIS=Barratt Impulsiveness Scale, UPPSP=UPPSP Impulsive Behaviors Scale, DDT=Delay-discounting Test, and *k* represents the discounting rate. ***p*<0.01, ****p*<0.001.
